# Evolutionary Dynamics of the *POTE* Gene Family in Human and Nonhuman Primates

**DOI:** 10.3390/genes11020213

**Published:** 2020-02-18

**Authors:** Flavia Angela Maria Maggiolini, Ludovica Mercuri, Francesca Antonacci, Fabio Anaclerio, Francesco Maria Calabrese, Nicola Lorusso, Alberto L’Abbate, Melanie Sorensen, Giuliana Giannuzzi, Evan E. Eichler, Claudia Rita Catacchio, Mario Ventura

**Affiliations:** 1 Department of Biology, University of Bari ‘Aldo Moro’, 70125 Bari, Italy; flavia.maggiolini@uniba.it (F.A.M.M.); ludovica.mercuri@uniba.it (L.M.); francesca.antonacci@uniba.it (F.A.); anacleriof@gmail.com (F.A.); Francesco.calabrese@uniba.it (F.M.C.); nico.lorusso27@gmail.com (N.L.); 2 Institute of Biomembranes, Bioenergetics, and Molecular Biotechnologies—National Research Council (IBIOM-CNR), 70125 Bari, Italy; a.labbate@ibiom.cnr.it; 3 Department of Genome Sciences, University of Washington School of Medicine, Seattle, WA 98195, USA; mels7@uw.edu (M.S.); ee3@uw.edu (E.E.E.); 4 Center for Integrative Genomics, University of Lausanne, 1015 Lausanne, Switzerland; giuliana.giannuzzi@unil.ch; 5 Howard Hughes Medical Institute, University of Washington, Seattle, WA 98195, USA

**Keywords:** gene family, evolution, primates, centromeres

## Abstract

*POTE* (prostate, ovary, testis, and placenta expressed) genes belong to a primate-specific gene family expressed in prostate, ovary, and testis as well as in several cancers including breast, prostate, and lung cancers. Due to their tumor-specific expression, *POTEs* are potential oncogenes, therapeutic targets, and biomarkers for these malignancies. This gene family maps within human and primate segmental duplications with a copy number ranging from two to 14 in different species. Due to the high sequence identity among the gene copies, specific efforts are needed to assemble these loci in order to correctly define the organization and evolution of the gene family. Using single-molecule, real-time (SMRT) sequencing, in silico analyses, and molecular cytogenetics, we characterized the structure, copy number, and chromosomal distribution of the *POTE* genes, as well as their expression in normal and disease tissues, and provided a comparative analysis of the *POTE* organization and gene structure in primate genomes. We were able, for the first time, to de novo sequence and assemble a *POTE* tandem duplication in marmoset that is misassembled and collapsed in the reference genome, thus revealing the presence of a second *POTE* copy. Taken together, our findings provide comprehensive insights into the evolutionary dynamics of the primate-specific *POTE* gene family, involving gene duplications, deletions, and long interspersed nuclear element (LINE) transpositions to explain the actual repertoire of these genes in human and primate genomes.

## 1. Introduction

Segmental duplications (SDs) are regions > 1 kbp in length sharing high sequence identity (>90%) and representing roughly 5% of the human genome [1,2]. SDs are enriched in pericentromeric and subtelomeric regions and due to the high sequence similarity between paralogous copies, undergo non-allelic homologous recombination causing structural rearrangements such as duplications, deletions, and inversions [3,4,5,6]. These recombination events are a source of genomic plasticity and variability that can directly increase the number of copies of the genes embedded within SDs [7]. Gene families specific to human and primate lineages have been recently detected, and the evidence of strong positive selection for some of these genes indicates that they may have played a substantial role in primate evolution [8,9,10,11].

*POTE* (prostate, ovary, testis, and placenta expressed) is the only multigene family coding for cancer-testis antigens (CTAs) or cancer-germline genes that show low expression in normal somatic tissues but are highly expressed in germ cells of the adult testis and fetal ovary, in prostate, and in placenta [12,13,14]. Moreover, *POTE* mRNA expression has been described in prostate cancer, and reverse transcription polymerase chain reaction (RT-PCR) experiments localized it in the epithelium of both normal and cancer prostate [15]. Based on their restricted expression in normal tissues and increased expression and association with poor prognosis in ovarian and other cancers, *POTEs* are potential biomarkers of cancer progression and therapeutic targets in these malignancies [16,17,18]. 

*POTE* is a primate-specific gene family consisting of 14 genes in human mapped on seven chromosomes divided into three phylogenetic groups [14,15,19,20,21]. Although purifying selection acts on this gene family (Ka/Ks ratio < 1), the Ka/Ks ratio between the human *POTE* genes is higher than the average reported for mammalian paralogs, suggesting that *POTE* genes are evolving faster than most others [15]. The *POTE* family originated from an ancestral ankyrin repeat domain 26 (*ANKRD26*) gene [20] most similar to the human *POTE* gene mapping on chromosome 8 (*POTEA*). The latter shares roughly 80% of sequence similarity with *ANKRD26* and it is the only *POTE* gene retaining the genomic segment that corresponds to exons 18–21 of *ANKRD26* [20]. A single *POTE* gene most similar to *POTEA* is found in marmoset, while *POTE* genes from two phylogenetic groups are found in the Old World monkeys (OWMs) [22]. Therefore, the *POTE* gene family was already present before the divergence of the New World monkeys (NWMs) and the OWMs, and the initial expansions that produced the three *POTE* gene groups must have occurred before the OWM—ape split (~20 million years ago), but after the OWM—NWM split [22]. Moreover, the presence of many different *POTE* genes on different chromosomes and their wide range of protein size (32 to 80 kDa) suggest that these individual genes may have evolved from an ancestral gene in response to different physiological demands [23].

In humans, all *POTE* genes have 11 exons in common. The overall size of the genes varies from ~32 to ~71 kb, depending mainly on the length of introns 6 and 9. All *POTE* genes contain a conserved 3′ UTR LINE-1 element, which promotes *POTE* dispersal in the primate genome [16], while all chromosome 2 *POTEs* (members of the *POTE* gene group 3), except for *POTEKP*, contain a C-terminal in-frame fusion with an actin gene as a result of a retrotransposition event in one of the ancestral *POTE* paralogs before the divergence of OWMs and apes. This insertion led to the formation of a new chimeric protein that contains both *POTE* and actin modules in the same protein [15,19,20,22,24]. Some members of the *POTE* family also include a long inverted repeat (LIR), described both in human and nonhuman primate genomes, which is able to form a large stem-loop and can play a role in expression variation [24]. *POTE* proteins include an N-terminal cysteine-rich region, five to seven ankyrin repeats made of tandemly repeated modules of ~33 amino acids each, and a C-terminal spectrin-like domain predicted to be mainly α-helical [15,22,23]. These features, together with the evidence of being associated with the plasma membranes, suggest that *POTE* proteins can be involved in protein-protein and cell membrane interactions [22]. 

Considering this intriguing functional and evolutionary data, we used the latest released sequencing data available for human and primate genomes to perform an in-depth genomic study of the *POTE* gene family. Using in silico analysis, molecular cytogenetics, and single-molecule, real-time (SMRT) sequencing of bacterial artificial chromosome (BAC) clones, we were able to provide a fine-scale description of human *POTE* alternative transcripts and trace the evolutionary history of *POTE* groups in primates. Moreover, our analyses identified a *POTE* tandem duplication in marmoset, which is totally collapsed in the reference genome, highlighting the need to improve the quality of current primate genome assemblies. This work will facilitate future functional studies on *POTE* genes and their potential involvement in cancer.

## 2. Materials and Methods

### 2.1. Retrieval of *POTE* Gene Entries in Human and Nonhuman Primates

Human *POTE* genes were identified from the Hugo Gene Nomenclature Committee (HGNC) database (https://www.genenames.org/), searching within the “gene groups” field. For nonhuman primates, the Ensembl database (https://www.ensembl.org/index.html; release 97–July 2019) was inspected. Human *POTE* genes were used to retrieve orthologs in other species: chimpanzee (*Pan troglodytes*, PTR), gorilla (*Gorilla gorilla gorilla*, GGO), orangutan (*Pongo abelii*, PPY), macaque (*Macaca mulatta*, MMU), and marmoset (*Callithrix jacchus*, CJA). The macaque gene ENSMMUG00000031128 was not reported as a human *POTEA* ortholog, but given that it shares the same Ensembl gene tree as *POTEA* (ENSGT00940000164614), it was included in the analysis (*Results*). The marmoset genes CH259-195P19_g1, CH259-195P19_g2 and CH259-106G20_g3 were predicted by Augustus (http://bioinf.uni-greifswald.de/augustus/) applying default parameters.

### 2.2. Features and Domain Prediction of *POTE* Transcripts

In order to investigate the domain content of the retrieved genes, protein sequences of each *POTE* transcript (for both human and nonhuman primates) were downloaded from Ensembl and analyzed with a combination of tools. The cysteine repeat content was investigated using the Protein Basic Local Alignment Search Tool (BLASTP, https://blast.ncbi.nlm.nih.gov/Blast.cgi) using as a query the cysteine repeat sequence reported in [15]. The presence of ankyrin repeats and β-actin retrogene was analyzed using the genomic mode of the Simple Modular Architecture Research Tool (SMART) (http://smart.embl-heidelberg.de/) searching also for PFAM domains, signal peptides, and internal repeats. Coiled-coil regions were retrieved from the Coils server (https://embnet.vital-it.ch/software/COILS_form.html) using the following parameters: window width 28, matrix MTIDK, and score threshold 0.5. Using the genomic sequences of the *POTE* genes, the presence of the LIR and of the β-actin retrogene out of the coding region were analyzed for each transcript via Blat [25].

### 2.3. FISH Analysis

The chromosomal distribution of the *POTE* paralogs in human and nonhuman primates was explored through fluorescence in situ hybridization (FISH). Metaphase spreads were obtained from *Homo sapiens* (HSA), *Pan troglodytes* (PTR), *Gorilla gorilla* (GGO), *Pongo pygmaeus* (PPY), *Macaca mulatta* (MMU), and *Callithrix jacchus* (CJA) fibroblast or lymphoblast cell lines. FISH experiments were performed using marmoset BAC clones and the following human fosmids: ABC12-49207800H13 (chr8:43292595-43332063, hg38), ABC12-46762300H6 (chr2:131226060-131265382, hg38), ABC8-41159000G3 (chr2:130096351-130133132, hg38), and ABC12-49059300N17 (chr18:14508900-14548830, hg38). Clones were directly labeled by nick-translation with Cy3-dUTP (PerkinElmer), Cy5-dUTP (PerkinElmer), and fluorescein-dUTP (Enzo) as described by [26] with minor modifications. Briefly, 500 ng of labeled probe was used for FISH experiments; hybridization was performed in 2×SSC, 50% (*v*/*v*) formamide, 10% (*w*/*v*) dextran sulphate, and 3 mg sonicated salmon sperm DNA in a volume of 10 mL. Post-hybridization washing was performed under high stringency conditions for human (three times 0.1×SSC at 60°C) and under low stringency conditions for chimpanzee, gorilla, orangutan, macaque, and marmoset (three times in 2×SSC at 37 °C and three times in 2×SSC, 50% formamide at 42 °C). Metaphases were simultaneously DAPI stained (200 ng/mL in 2×SSC, 5 min incubation). Fluorescence signal intensity from DAPI, Cy3, and Cy5 was detected with specific filters using a Leica DMRXA2 epifluorescence microscope (Leica Microsystems GmbH, Wetzlar, Hesse, Germany) equipped with a cooled CCD camera. Digital images were separately recorded as grayscale pictures and subsequently pseudocolored and merged using Adobe Photoshop software (Adobe, San Jose, California, United States).

### 2.4. Radioactive Screening on High-Density Filters

Due to the absence of FISH signals on marmoset chromosomes, we performed a radioactive BAC library screening to collect marmoset clones putatively containing the *POTE* sequence. The radioactive genomic hybridization of the marmoset CHORI-259 BAC library (segment 1, 5.1× coverage) was carried out according to the protocol available at CHORI BACPAC resources (https://bacpacresources.org/highdensity.htm). Two different probes (STS1 and STS2) were drawn on the latest marmoset assembly (WUGSC 3.2/calJac3) in the region where the human RefSeq *POTE* genes are mapped and labeled with ^32^P. Probe STS1, 425 bp in length, was designed on the region spanning the first exon-intron junction (chr16:10145382-10145806) and amplified through the following primer pair: F-CGCCCCGACCTCTGAGTTTT/R-AGCCATCTGCTCCCCCTTTG. Probe STS2, 431 bp in length, was designed on the eighth intron-exon junction (chr16:10127122-10127552) and amplified through the primer pair F-ACCACAGCCAGGAGCGACAC/R-CCACAAGGCAAATGGGGTCA. In order to remove false positives, a PCR assay was carried out with the same primers on the positive BAC clones. The PCR was performed as follows: 4 min initial denaturation at 94 °C, followed by 35 cycles of 95 °C for 30′, 60 °C for 30′, and 72 °C for 1′. Final extension was at 72 °C for 5′. The reaction mixture consisted of 0.5 μL dNTPs (2 mM), 1 μL each primer (10 μM), 0.3 μL Taq DNA polymerase (5 U/μL), 0.5 μL MgCl2 (50 mM), 2.5 μL 10× reaction buffer (10×), 3μL of DNA template (50 ng/μL), and water up to 25 μL.

### 2.5. PacBio Callithrix Jacchus BAC Sequencing and Assembly

Marmoset DNA from the BAC clones selected by high-density filter library screening was extracted, pooled, prepared as a PacBio SMRTbell library (Pacific Biosciences of California, Inc, Menlo Park, California, United States), sequenced to high coverage, and assembled as described previously [27]. Briefly, PacBio libraries were prepared using the PacBio SMRTbell Template Prep 1.0 kit, size selected, and sequenced on the PacBio RS II platform with P6-C4 chemistry. The sequences were assembled with Canu v1.5 [28], polished with Quiver, and examined for misassembly by visualizing the read depth of PacBio reads in Parasight (https://baileylab.brown.edu/parasight/), using coverage summaries generated during the resequencing protocol.

### 2.6. Phylogenetic and LINE Analyses

Phylogenetic analysis of the *POTE* genes was conducted by the maximum likelihood method. The sequences used to build the tree were the regions mostly conserved and devoid of sequence gaps shared between all human and nonhuman *POTE* genes. The timetrees shown were generated using the RealTime method [29]. Divergence times for all branching points in the topology were calculated using the maximum likelihood method based on the Jukes-Cantor model [30]. Evolutionary analyses were conducted in MEGA7 [31]. The LINE content of each *POTE* gene was explored and annotated based on RepeatMasker, version open-4.0.9 (http://www.repeatmasker.org/).

### 2.7. Gene Expression

In order to retrieve data on the differential expression of *POTE* genes in human cancers, raw data (.fastq files) from mRNA-sequencing studies on breast cancer subtypes (PRJNA227137) and whole transcriptome profiling of esophageal adenocarcinoma and Barrett’s esophagus (PRJEB11797) were downloaded from the European Nucleotide Archive (https://www.ebi.ac.uk/). Kallisto was used for the pseudoalignment and transcript quantification against the hg38 transcriptome with the following parameters: -b 50, --rf-stranded for PRJEB11797; -b 50 for PRJNA227137 [32]. In order to assess the expression profile of *POTE* transcripts, we performed six different comparisons (HER2-positive, non-triple-negative, triple-negative breast cancers, Barrett’s esophagus with low-grade dysplasia and esophageal adenocarcinoma vs. normal, and esophageal adenocarcinoma vs. Barrett’s esophagus non-dysplastic), and we identified differentially expressed genes (FDR *q* value < 0.05) using Sleuth [33].

### 2.8. Estimating the Timing of Human-Specific *POTE* Duplications

With the aim of dating the human-specific expansion of *POTE* genes, multiple sequence alignments (MSAs) of human *POTE* paralogs and orthologs from human, chimpanzee, and orangutan were generated using Clustal W [34]. We constructed a series of phylogenetic trees using the neighbor-joining method with a complete deletion option (MEGA7) [31]. Genetic distances were calculated using the Kimura 2-parameter with standard error estimates (an interior branch test of phylogeny; N = 500 bootstrap replicates); Tajima’s relative rate test was used to assess the validity of the molecular clock. We then estimated the divergence time using the equation T = K/2R and an estimated divergence time of 6 million years between human and chimpanzee and 16 million years between human and orangutan.

## 3. Results

### 3.1. *POTE* Gene Family in the Human Genome

**Figure 1:**
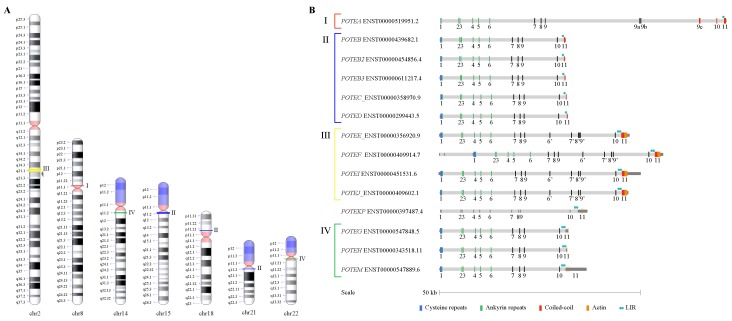
(**A**) Chromosome ideograms showing the localization of *POTE* (prostate, ovary, testis, and placenta expressed) genes in human. The colored bars indicate the four different *POTE* groups described later on in the results: red for group I, blue for group II, yellow for group III, and green for group IV. *POTEKP* mapping is indicated by the gray arrowhead. (**B**) Domain structure of the 14 canonical transcripts of human *POTE* genes. Coding (colored vertical bars) and noncoding (shorter and gray vertical bars) exons are shown and numbered on each transcript and are based on the Ensembl gene sequences. Half-sized LIR is indicated by a single light blue circle. Dark gray blocks flanking the transcripts indicate UTRs. Orange framed rectangles indicate actin-coding sequences outside of the CDS.

The latest update (2019-11-27) of the HUGO Gene Nomenclature Committee (HGNC) database [35] was inspected to retrieve the annotated members of the *POTE* gene family in the human genome: 14 *POTE* paralogs, ranging in size from 30.9 to 71 kb and distributed on seven chromosomes, were found (Table 1 and Figure 1A). The sequence and structure of each gene were then analyzed via the Ensembl genome database [36], showing that most *POTE* genes in human encode for different proteins or produce long noncoding RNA (up to 5 alternative transcripts per gene, Table 1). All *POTE* genes contain the same 11 exons; furthermore, *POTEA* comprises three paralog-specific exons (9a to 9c, between exons 9 and 10), four paralogs (*POTEE*, *POTEF*, *POTEI*, *POTEJ*) contain an extra copy of the exons 6 to 9 (named 6′ to 9′), and the *POTEF* canonical transcript starts with two extra and paralog-specific exons (Figure 1B).

For further insight into the domain content of the human *POTE* paralogs, we analyzed each Ensembl-predicted protein through a combination of the following tools: SMART [37], BLASTP, and the Coils server [38]. The β-actin retrogene insertion described by Lee and colleagues [22] at the carboxyl terminus of the *POTE* genes was shown to be present in eight out of 14 genes, and in four cases was in-frame, resulting in an actin module in the same *POTE* protein. Cysteine and ankyrin repeats and coiled-coil domains were also annotated in detail. Moreover, we searched the *POTE* sequences for the presence of the LIR in the intron between exons 10 and 11. Eight genes contained a full-sized LIR sequence while six carried a half-sized LIR sequence (Table 2, Table S1 and Figure S1).

**Figure 2:**
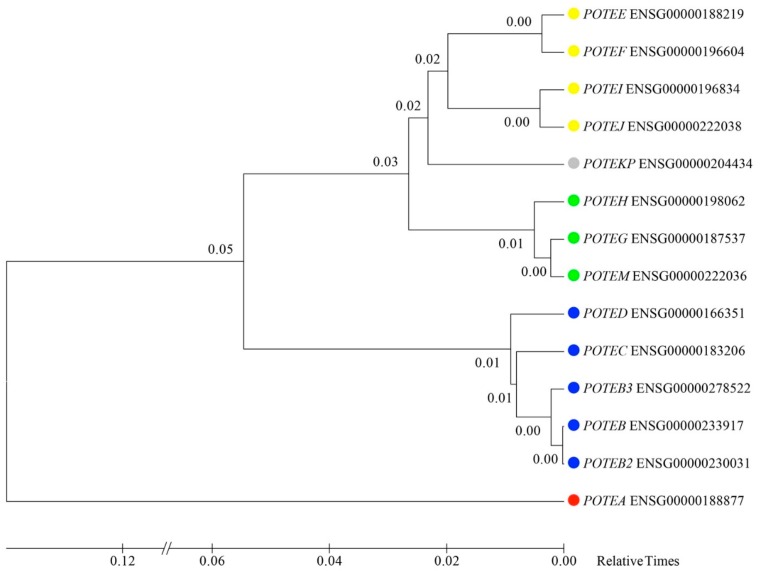
Phylogenetic tree built using the Ensembl genomic sequence of the 14 human *POTE* genes from exons 1 to 3. For each paralog, the Ensembl ID is indicated together with the approved symbol. Colored circles indicate the four different groups: red for group I, blue for group II, yellow for group III, and green for group IV. The estimated log likelihood value of the topology shown is −19,591.8948. The tree is drawn to scale, with branch lengths measured in the relative number of substitutions per site.

A maximum likelihood phylogenetic tree using exon 1 to exon 3 genomic sequences was constructed to understand the evolutionary relationship between the 14 genes. Based on the inferred phylogenetic tree and on the above-mentioned analyses, we classified the human *POTE* genes into four different groups (instead of the three previously reported): group I contains only *POTEA*; group II is comprised of *POTEB*, *POTEB2*, *POTEB3*, *POTEC*, and *POTED*; group III contains *POTEE*, *POTEF*, *POTEI*, and *POTEJ*; group IV has *POTEH*, *POTEG*, and *POTEM*. *POTEKP* shows features of both groups III and IV and cannot be unambiguously classified (Figure 2).

### 3.2. *POTE* Expression Levels in Cancer

We performed comparative analyses of several cancer tissues vs. normal tissues in order to detect a statistical difference in the gene expression of *POTE* genes. Using Kallisto–Sleuth analyses, we revealed different isoforms of the *POTE* gene that was differentially expressed in the tested conditions. In detail, we detected an up-regulation of transcripts ENST00000451531.6 (*POTEI*) and ENST00000511306.5 (*POTEC*) in esophageal adenocarcinoma. The comparisons of HER2-positive breast carcinoma, triple-negative breast cancer, and non-triple-negative breast cancer versus normal human breast organoids disclosed an up-regulation of nine *POTE* transcripts: ENST00000624267.3 (*POTEB3*), ENST00000511306.5 (*POTEC*), ENST00000358087.9 (*POTEE*), ENST00000361163.8 and ENST00000409914.6 (*POTEF*), ENST00000451531.6 and ENST00000652235.1 (*POTEI*), ENST00000397487.3 (*POTEKP*), and ENST00000552966.5 (*POTEM*) (Table S2).

### 3.3. *POTE* Gene Family in Primate Genomes

In order to investigate the distribution of the *POTE* genes among primate genomes, we adopted a two-pronged approach. First, we used the 14 human genes as a query in Ensembl and retrieved the corresponding orthologs for the following species: chimpanzee, gorilla, orangutan, macaque, and marmoset. Next, we tested, using FISH, four human fosmid clones containing *POTE* sequences on the same primate species, including human.

The Ensembl inspection allowed the identification of five *POTE* genes in the PTR genome, three in GGO, four in PPY, three in MMU, and one in CJA (Table 3 and Table S3). Two out of five PTR genes were already annotated in Ensembl with the *POTEA* and *POTEB* gene symbols, and one out of four PPY genes was annotated with the *POTEA* symbol, while for the other species, none of the genes was annotated with any of the *POTE* gene symbols. Although a majority (10/16, 62.5%) of the primate-predicted genes contained gaps in their sequences, we analyzed them in terms of alternative transcripts, structure, and domain, mirroring what was done for human. Both the half- and full-sized LIR and the β-actin retrogene were found in chimpanzee, gorilla, and orangutan, while the macaque and marmoset genes were truncated and thus were not informative (Table 3, Table S3 and Figure S2). The predicted *POTE* genes mapped in chimpanzee, gorilla, and orangutan on the chromosomes orthologous to HSA8q and HSA15q; in chimpanzee and orangutan, on the orthologs of HSA14q; in chimpanzee, gorilla, orangutan, and macaque, on chromosomes orthologous to HSA2q; in macaque, on the ortholog of HSA20; in marmoset, on an unassembled contig (Table 3 and Table S3).

**Figure 3:**
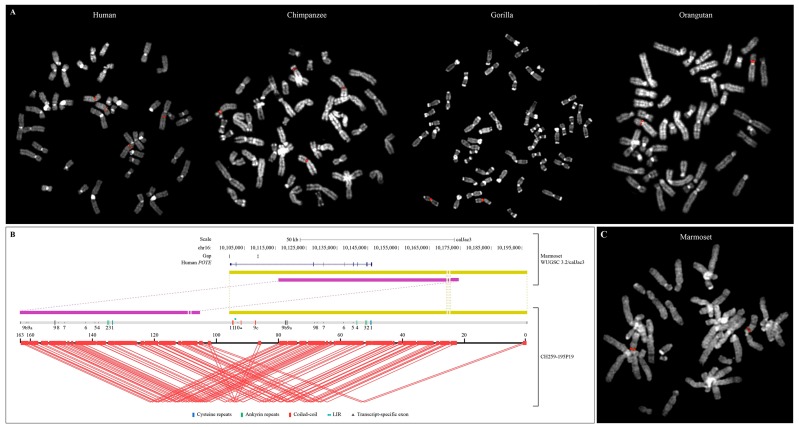
(**A**) FISH validation of the fosmid clone ABC12-49207800H13 (in red) in human, chimpanzee, gorilla, and orangutan. (**B**) Parasight view of the CJA BAC CH259-195P19. Red lines show internal duplications. The gray bar indicates the *POTE* gene content, with coding (colored) and noncoding (dark gray) exons shown as vertical bars. Yellow and purple blocks show the most continuous portion (96 kb) of the marmoset reference genome where the BAC maps. The human *POTE* RefSeq mapping is shown in blue. (**C**) FISH results of the clone CH259-195P19 (in red) in marmoset.

The localization of the *POTE* genes in primate genomes was then cytogenetically confirmed by using human clones mapping on *POTEA* (ABC12-49207800H13), *POTEC* (ABC12-49059300N17), *POTEE* (ABC12-46762300H6), and *POTEF* (ABC8-41159000G3) as probes in FISH experiments on the metaphases of one individual for each of the six analyzed species, plus human (Table 4 and Figure 3A). Unfortunately, no hybridization signals were detected on marmoset chromosomes likely as a result of higher sequence divergence between the human clones and the marmoset genome. Because of the high sequence identity among *POTE* paralogs, cross hybridization signals were detected in all the other species with all the probes except for the one containing *POTEA*. Indeed, ABC12-49207800H13 showed hybridization signals only on HSA8 in human, chimpanzee, gorilla, and orangutan and on HSA2q on human and chimpanzee, indicating that the *POTE* gene on chromosomes orthologous to HSA8 is the most divergent.

FISH results confirmed the localization of the Ensembl-predicted genes in chimpanzee, gorilla, orangutan, and macaque. In addition, hybridization signals were also reported on chimpanzee, gorilla, and orangutan HSA18; chimpanzee and orangutan HSA21; gorilla HSA13 and HSA14; orangutan HSA2p; and macaque HSA10. All the signals mapped pericentromerically, except for HSA2q in human and macaque (Table 4).

Due to the lack of mapping information of *POTE* genes in marmoset (both from cytogenetics and in silico data), species-specific BAC clones containing the *POTE* sequence were retrieved through hybridization screening of high-density BAC filters. Two customized probes were designed corresponding to the first exon/intron junction and to the eighth intron/exon junction (named STS1 and STS2, respectively; *Materials and Methods*) using the sequence from the WUGSC 3.2/calJac3 marmoset reference assembly, orthologous to the human *POTEA*. Twenty BAC clones were positive for both STS1 and STS2 and were mapped by FISH on marmoset chromosomes: 11 out of 20 mapped to HSA8; one clone mapped to both HSA8 and HSA7, one to HSA8 and HSA10, and the other seven had signals detected on HSA2q, HSA7, and HSA13 (Table S4).

Four of these clones (CH259-195P19, CH259-106G20, CH259-281G20, and CH259-156L15 mapping on HSA8, HSA8 and HSA7, HSA2q, and HSA13, respectively) were PacBio sequenced, and the insert sequences de novo assembled [39]. The assembled clones, ranging in size from 163 to 189 kb, were mapped by BLAST against the latest release of the marmoset reference genome (WUGSC 3.2/calJac3) and confirmed the cytogenetic localization: CH259-195P19 and CH259-106G20 mapped on chromosome CJA16 (HSA8), CH259-156L15 on chromosome CJA5q (HSA13), and CH259-281G20 on chromosome CJA6 (HSA2q). The extra signal observed by FISH on HSA7 with clone CH259-106G20 might be due to a duplication of a small region of the BAC that is collapsed and absent in the marmoset reference genome. Interestingly, the length of the obtained clones strongly disagreed with the spanned sequence on the marmoset reference genome in 2/4 cases (Table S4). Moreover, the gene content of the four BACs was inspected by Augustus [40], revealing one to eight genes per clone (Figure S3). Nevertheless, the genes predicted in the sequences of CH259-156L15 and CH259-281G20 showed no similarity with the *POTE* genes in terms of sequence or structure. The positivity of these clones to the library screening might be due to the presence of an undetected small duplication of a fragment of *POTE* sequences on marmoset chromosomes HSA2q and HSA13. Clone CH259-195P19 instead contained two copies of the *POTE* gene (hereinafter referred to as CH259-195P19_g1 and CH259-195P19_g2), while CH259-106G20 contained a truncated copy of a *POTE* gene (hereinafter referred to as CH259-106G20_g3) (Table S5, Figure S2 and Figure 3B,C). Further inspection of the CH259-195P19 sequence with the program Parasight revealed a tandem duplication within the clone itself (Figure S2 and Figure 3B). A BLAST analysis between clones CH259-106G20 and CH259-195P19 revealed that the two clones partially overlap and the truncated *POTE* gene CH259-106G20_g3 corresponds to CH259-195P19_g1; therefore it was not considered for further analysis. Sequence and structure analysis of CH259-195P19_g1, CH259-195P19_g2, and the marmoset annotated gene ENSCJAG00000033335 revealed that the latest does not correspond to either one of the two PacBio sequenced genes (Figure S2). Nevertheless, gene ENSCJAG00000033335 contains several gaps, is truncated, and is annotated on an unassembled contig; therefore, the differences between the annotated gene and the PacBio-sequenced genes could be an artifact due to a marmoset reference genome misassembly. The presence of a third copy of the *POTE* gene in the marmoset genome cannot be confidently inferred.

### 3.4. Structure of the Intraduplication of *POTE* Genes

The *POTE* group III, mapping on chromosome 2q in human, is composed of genes containing a duplication of exons 6 to 9 (named 6′ to 9′). The same duplication has been found in PTR (ENSPTRG00000024151 and ENSPTRG00000022897, both on HSA2q), GGO (ENSGGOG00000007645 on HSA2q), and PPY (ENSPPYG00000012746 on HSA2q). To study the evolution of this duplication, relative to the separation of groups III and IV from their common ancestor gene, we inferred two phylogenetic trees: the first using the whole region doubled in group III genes (exons 6 to 9 and their duplication) extracted from the human *POTE* genes (Figure S4A); the second using the short sequence comprising exons 6 and 7 and the intron between them, extracted from all the available sequences (Figure S4B). Also, in this case, the analyzed sequence was chosen based on the largest sequence available in the studied species: exons 6 and 7 were the only ones (among the duplicated exons) also present in the macaque sequences (Figure S2). Both trees showed that the duplication likely originated from the ancestor of *POTEKP*.

### 3.5. Repetitive Elements in *POTE* Genes

**Figure 4:**
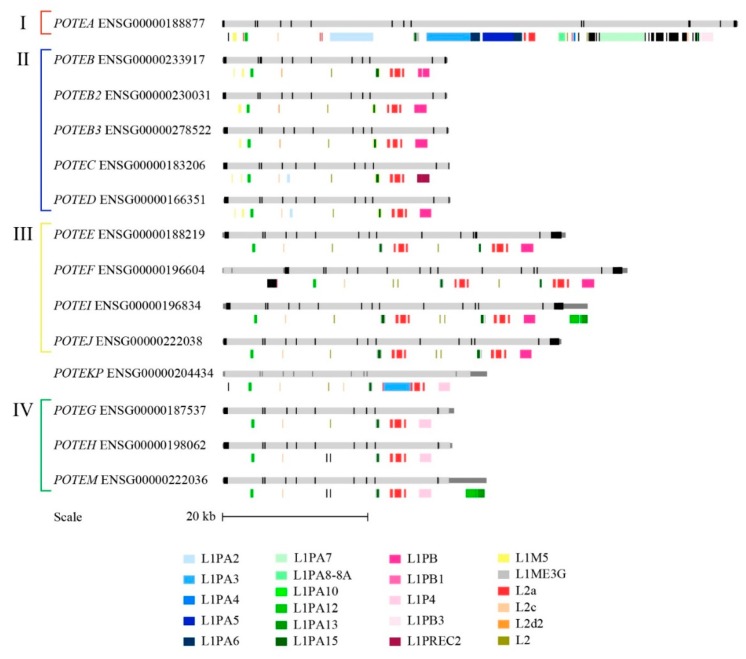
LINE contents of the 14 human *POTE* genes are displayed under each gene representation. The LINEs annotated as black blocks represent less frequent elements, thus not explained in the color legend at the bottom of the figure.

Since the expansion of LINE-1 retrotransposons has been finely dated [41], we decided to use the LINE content in the *POTE* genes as additional information to reconstruct the evolutionary history of the gene family. We thus inspected the human *POTE* gene loci for LINE content (Figure 4) and noticed that it is clearly related to the *POTE* group classification: (i) group I has been specifically and massively invaded by L1PA elements, (ii) group I and group II share the presence of an L1M5 element in the first intron, which is absent from groups III and IV; (iii) group II shows the cluster L1M5-L1PA15-L1M5 in the ninth intron, which in all other groups is composed of L1ME3G-L1PA15-L1ME3G; (iv) groups III and IV display a very similar LINE composition, except for the content of the exon 6 to 9 region, which is duplicated in group III. This organization was mostly confirmed by a similar analysis performed on the retrieved primate genes (Figure S5). In addition, the LINE analysis in primates revealed that ENSPTRG00000050347, although not displaying any of the duplicated exons (likely because of the gaps), also shows a duplicated LINE content between exons 9 and 10, similar to group III genes.

### 3.6. Inferring the Evolutionary Relationships of *POTE* Paralogs

**Figure 5:**
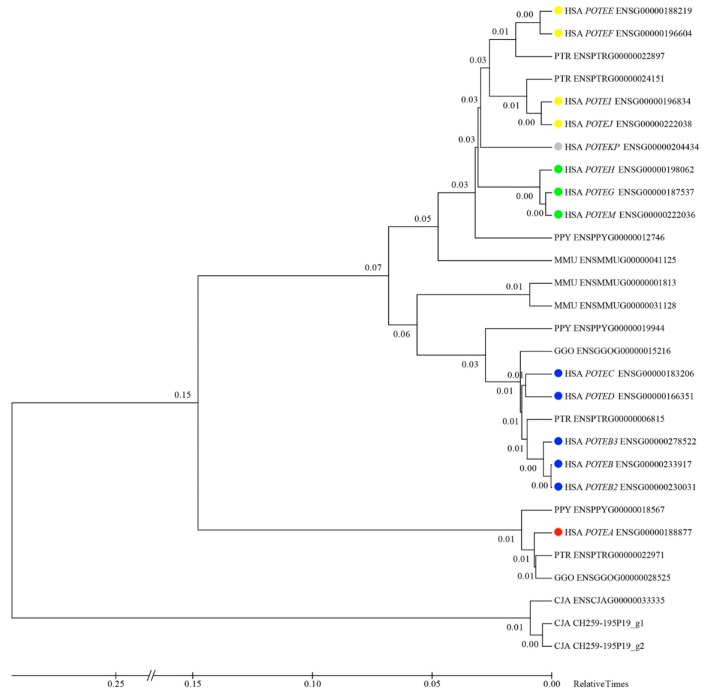
Phylogenetic tree built using the Ensembl genomic sequence from exons 1 to 3 of the collected human and nonhuman primate *POTE* genes. CH259-195P19_g1 and CH259-195P19_g2 sequences are based on the Augustus prediction; all the others have been retrieved from the Ensembl browser. For each paralog, the Ensembl ID is indicated together with the approved symbol. Colored circles indicate the four different groups: red for group I, blue for group II, yellow for group III, and green for group IV. The estimated log likelihood value of the topology shown is −74,555.8895. The tree is drawn to scale, with branch lengths measured as the relative number of substitutions per site.

To investigate the evolutionary dynamics of the four *POTE* groups, a maximum likelihood phylogenetic tree using the retrieved genomic sequences (Ensembl genes plus the ones predicted by Augustus in the CJA clones) for the mostly conserved regions and devoid of sequence gaps among all *POTE* genes (exons 1 to 3) was built (Figure 5). Three genes (ENSPTRG00000050347, ENSGGOG00000007645, and ENSPPYG00000005548) were not included because their sequence is not complete in the analyzed region. The tree, together with sequence structure and domain content data, FISH experiments, and LINE content, allowed the designation of the different primate genes to a specific *POTE* group. Hence, we found evidence of group I genes in PTR (ENSPTRG00000022971), GGO (ENSGGOG00000028525), PPY (ENSPPYG00000018567) and CJA (ENSCJAG00000033335, CH259-195P19_g1 and CH259-195P19_g2); group II genes in PTR (ENSPTRG00000006815), GGO (ENSGGOG00000015216) and PPY (ENSPPYG00000019944); and group III genes in PTR (ENSPTRG00000024151 and ENSPTRG00000022897). The sequences of one orangutan (ENSPPYG00000012746) and three macaque genes (ENSMMUG00000001813, ENSMMUG00000031128 and ENSMMUG00000041125) are either truncated or interrupted by gaps (outside of the region used to generate the phylogenetic tree); this is the reason why, in these cases, the Ensembl mapping seems to disagree with the evolutionary tree and they could not be confidently assigned to a specific *POTE* group.

We assigned to specific groups the macaque and orangutan genes looking at the LINE content and evolutionary time coupled to the phylogenetic tree. In detail, ENSPPYG00000012746 displays features of both group III (Ensembl mapping and LINE contents) and IV (annotated on Ensembl as the orangutan ortholog of human group IV genes) genes; we can hypothesize that it represents an ancestral form of these two human groups (as shown by the evolutionary tree). ENSMMUG00000001813, shows the LINE contents (L1M5 in the first intron) and the phylogenetic tree clustering of *POTE* group II genes; the ENSMMUG00000031128 LINE content is not clearly indicative of a *POTE* group (it might have selectively deleted L1M5 in the first intron) and shows the phylogenetic tree clustering of *POTE* group II genes. Thus, although these genes were originally retrieved as potential orthologs of human group I *POTEs*, these data suggest that they may be better classified as group II genes. Similarly, ENSMMUG00000041125 shows the LINE content of *POTE* group II genes (L1M5 in the first intron), but in the phylogenetic tree seems to cluster with groups III and IV genes. Since L1M5 is an element that colonized the genomes of Eutherians (thus more than 100 million years ago) and was already present among *POTE* genes before the radiation of the three primate groups, this gene could represent an ancestor of groups III and IV (Figure S5).

### 3.7. Timing of Human-Specific *POTE* Duplications

To estimate the timing of the human-specific *POTE* gene duplications during evolution, we constructed a series of phylogenetic trees and estimated the divergence time using molecular clocks and predicted divergence time of 6 million years between human and chimpanzee and 16 million years between human and orangutan.

Phylogenetic analysis of the group II *POTE* genes predicts that a first duplication occurred 1.07 million years ago (Mya) (±110 thousand years ago), giving rise to the second *POTE* copy on human chromosome 15 (harboring *POTEB3* and the ancestor of *POTEB* and *POTEB2*) (Figure S6), followed by a second event of duplication that occurred 550 thousand years ago (±80 thousand years ago) generating *POTEB* and *POTEB2* genes (Figure S6).

Phylogenetic analysis of the group III *POTE* genes predicts that the oldest human specific duplication events include a first round of duplicative transposition that occurred 1.9 Mya (±146 thousand years ago) involving *POTEE* and *POTEF* (Figure S6) and a second duplication event that occurred 1.57 Mya (±133 thousand years ago) involving *POTEI* and *POTEJ* (Figure S6).

Finally, phylogenetic analysis of the group IV *POTE* genes predicts that a first duplication occurred 1.06 Mya (±135 thousand years ago) for *POTEH* and the ancestor of *POTEG* and *POTEM* (Figure S6) and a second duplication occurred 440 thousand years ago (±87 thousand years ago), giving rise to *POTEG* and *POTEM* (Figure S6).

## 4. Discussion

The *POTE* gene family was first identified more than 15 years ago [15] and since then it has gained more and more interest in the scientific community for being a candidate for cancer-targeted therapy, as well as for showing signs of purifying selection. In this study, we performed a detailed characterization of the organization and gene structure of the *POTE* genes among human, great ape (chimpanzee, gorilla and orangutan), OWM (macaque), and NWM (marmoset) genomes and classified the full extent of primate *POTE* genes in four groups, instead of the previously suggested three [15,20]. Based on our data, the *POTE* gene family ranges from two copies in the marmoset genome, to at least four paralogs in macaque, six in gorilla, seven in both orangutan and chimpanzee, and 14 in human. Interestingly, the human specific expansion has been mediated by several rounds of duplications, all of which occurred in the last two million years. This increase of gene copies is consistent with the overall expansion of SD in the ancestral great ape lineage [43]. Our data suggest that *POTE* duplication evolved in a tandem organization in NWM (e.g., marmoset) and subsequently dispersed in human and great apes lineages as has been reported for other SDs [44,45].

*POTE* paralogs and transcripts had specifically been found expressed in commonly occurring cancers (including lung, colon, breast, pancreas, and ovary) and/or normal tissues [17]. Real-time PCR, microarray analyses, and recent ‘omic’ data revealed *POTE* expression profiles associated with pathogenic conditions and the possible involvement of *POTE* proteins in robust detection of disease progression [46]. We inspected publicly available resources in order to compare the expression levels of each paralog in cancer tissues in human and, in agreement with previous data [17], we found an overall overexpression of *POTE* paralogs belonging to groups II, III, and IV in cancer, and more specifically, in breast cancer and esophageal adenocarcinoma. This is intriguing, since *POTE* proteins, and more specifically, POTE-actin fusion proteins (i.e., group III), had been clearly associated with pro-apoptotic effects [46] and it had been proposed that the pro-apoptotic role is carried out through the interaction of *POTE* proteins with the cytoskeleton mediated by the fusion of actin [22].

The *POTE* gene family amplification process has been considered to be primate specific and, as generally acknowledged, our study confirms it occurred in the OWM and NWM common ancestor. Indeed, besides the 14 human paralogs, we integrated literature with data retrieved from the most recent assemblies of five primate genomes, cytogenetic assays, high-density filter BAC library screening, and SMRT long-read sequencing, identifying a total of 26 primate *POTE* genes (of which 18 were annotated on the primate genome references and eight were identified by FISH). All retrieved *POTE* genes map within 500 kb and 1 Mb from the centromeres in primate chromosomes, except for the ortholog of HSA2q in human and macaque. However, in both species, the *POTE* locus on 2q corresponds to an ancestral centromere, which was inactivated following a chromosomal fusion (in the human lineage) or a neocentromere emergence (in the OWM ancestor) [47,48,49].

Notably, all human centromeres harboring the *POTE* genes co-localize with the SF2 family of alpha satellite DNA [15], and this leads us to hypothesize that *POTE* genes may have been copied and dispersed by the same centromeric exchanges that created the alphoid suprachromosomal families in great apes [50,51,52]. Also, *POTE* genes must have subsequently colonized HSA14 and HSA15 pericentromeric regions in the ancestor of the great apes as they are absent from the orthologous regions in macaque and marmoset [53]. Interestingly, these two chromosomes evolved as the result of a chromosomal fission that occurred in the ancestor of the great apes. In other words, prior to the evolutionary fission, these two regions were not pericentromeric and likely only became targets for *POTE* duplications after they became pericentromeric, supporting the long-held view of an intimate association between African ape pericentromeric regions and interspersed duplications [54]. It is noteworthy that the genomic organization of the *POTE* gene family shows some peculiarity when compared to other human- and primate-specific gene families such as the neuroblastoma breakpoint family (*NBPF*) [55], the nuclear pore complex interacting protein (*NPIP*) [56], and the leucine-rich repeat-containing protein 37 (*LRRC37*) [9]. *POTE* members map pericentromerically on several chromosomes as interchromosomal duplications, while *NBPF*, *NPIP*, and *LRRC37* have multiple copies on a single chromosome (human chromosome 1, 16, and 17, respectively), displaying only a pattern of intrachromosomal duplications. This difference is most likely related to the pericentromeric location of the ancestral copy of the *POTE* genes and the pericentromeric-directed mechanism for non-homologous interchromosomal exchange during evolution [57].

In this study, we have performed a detailed analysis of the LINE content in order to reconstruct the evolution of the *POTE* genes in human and nonhuman primates. Our analysis identified an L1PA2 insertion in the ninth intron of *POTEA* in human and chimpanzee. These elements date 7 million years (Myr) [41], and thus the emergence of this insertion in the common ancestor of human and chimpanzee is consistent with the split of the two lineages, estimated around 5 Myr ago [58]. Another L1PA2 insertion is displayed on the fourth intron of human *POTEC* and *POTED*. This could either indicate that it was already present in the ancestor of group II genes and underwent a deletion in the group II genes on HSA15 (*POTEB*, *POTEB2* and *POTEB3*) or that it was specifically inserted in an ancestor gene of *POTEC* and *POTED*. The second hypothesis is the most parsimonious and is also supported by the fact that there is no trace of L1PA2 insertion in the chimpanzee group II gene (ENSPTRG00000006815). Moreover, we found insertions of an L1PA3 element in human, chimpanzee, gorilla, and orangutan *POTEA* genes, in the region containing exon 9a, 9b, and 9c (the *POTEA*-specific exons); L1PA3 is dated to 31 Myr [41]; thus, its insertion in *POTEA* must have occurred between 13 and 31 Myr ago. L1PA3 is also present in human *POTEKP* and in the orangutan gene ENSPPYG00000019944; however, the insertion locus is different, suggesting that these are independent insertions. 

**Figure 6:**
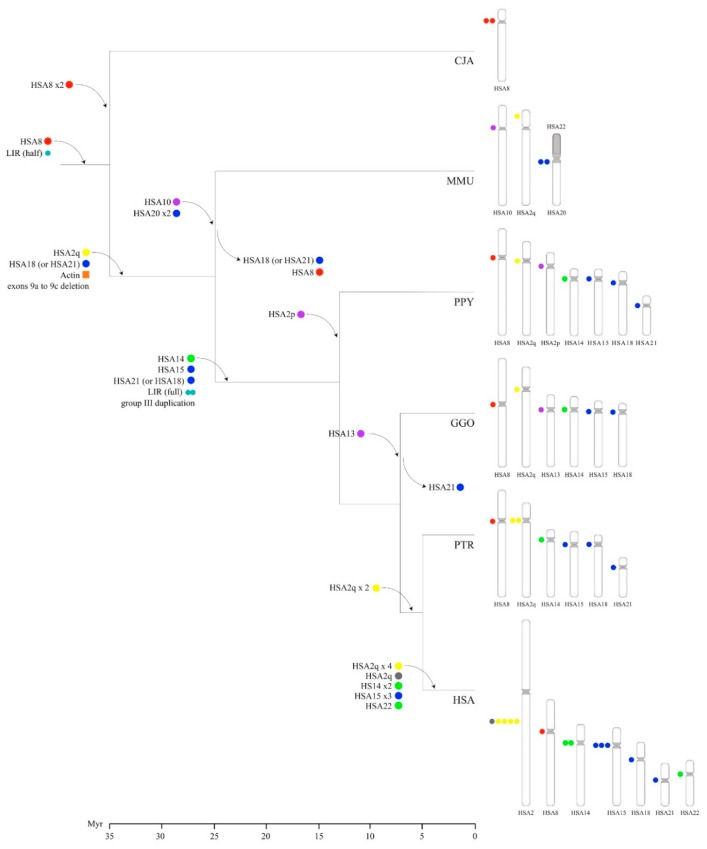
Evolutionary model of the *POTE* gene family. The emergence of each *POTE* copy, the actin module fusion, the full-sized LIR, and the group III intraduplication are described. Colored circles indicate the four different groups: red for group I, blue for group II, yellow for group III, and green for group IV. Purple circles represent *POTE* paralogs that have not been assigned to specific groups due to the lack of sequence data.

Combining this information with phylogenetic data and a detailed characterization of the total number of exons, the presence of a half/full-size LIR and of the β-actin retrogene, we generated the following evolutionary model (Figure 6). The *POTE* gene family emerged in the primate genomes on the chromosome orthologous to HSA8 (*POTEA* ancestor gene) before the NWM divergence (>35 Myr) as a direct descendant of *ANKRD26* with which it shares 14 exons [22]. It then underwent subsequent rounds of duplication and lineage-specific deletions that created the current census of gene family members. The first *POTE* gene (*POTEA*) underwent a tandem duplication in the marmoset genome and, to date, these two genes represent the only full known *POTE* complement in this nonhuman primate. Phylogenetic data and LINE analysis suggest that groups II and III emerged in the ancestor of macaque and great apes, after a deletion event that removed exons 9a to 9c and occurred in the ancestor of group II and III genes. Further rounds of duplication created additional copies of group II *POTEs* in the ancestor of the great apes that led to the current group II complement on HSA15, HSA18, and HSA21. In addition, the human lineage experienced a further intrachromosomal duplication that created three copies of the *POTE* gene on chromosome HSA15 (*POTEB*, *POTEB2*, and *POTEB3*) (Figure 6).

The actin module fused, likely on chromosome HSA2q, with one *POTE* gene in the common ancestor of OWM and the great apes [22], creating the ancestor of group III and IV genes. Then, this gene was, in turn, copied on HSA14 in the ancestor of the great apes, to create the first of the group IV genes. There, a frameshift mutation created a stop codon that caused a premature termination before the actin module [22]. Likely, this mutation is also responsible for the absence of the coiled-coil domains in group IV proteins. Moreover, in the great ape ancestor, the region containing *POTE* exons 6 to 9 underwent an intragenic duplication (group III duplication) in the chromosome HSA2q gene. Notably, our phylogenetic analysis shows that the duplicated region distinguishing the current group III genes (exons 6′ to 9′) is more similar to the exon 6 to exon 9 region of the genes from group IV and *POTEKP* than to the paralogous region of group III *POTEs*. This may be explained by two different scenarios: (i) the duplication of exons 6–9 was created by an intragenic duplication followed by gene conversion events that made it more similar to group IV genes, (ii) or it was created by an intergenic duplication that involved the ancestor of group IV genes (Figure 6).

The LIR in the last *POTE* intron of the genes belonging to groups III and IV has been associated with overexpression [24]. The full structure of the LIR was previously reported to have emerged in the common ancestor of chimpanzee and human [24]. Nevertheless, we have found evidence of the presence of the full-sized LIR also in gorilla and orangutan *POTE* genes, suggesting that it originated earlier in the great ape ancestor (Figure 6).

In our model, the *POTE* gene family has been created in primate genomes by both concerted and birth-and-death evolutionary models [59]. This allowed the creation of the four *POTE* groups in all the analyzed genomes and facilitated functional differentiation of *POTE* proteins [20,23]; at the same time, in our study, we have reported lineage-specific duplication/deletions. 

Another interesting finding of this work is that through SMRT sequencing and assembly of four marmoset BAC clones, we were able to detect and correct two regions that were misassembled in the marmoset reference genome (WUGSC 3.2/calJac3). More specifically, the marmoset *POTE* locus on chromosome 16 is devoid of nearly 67 kb of sequence containing one copy of *POTE* genes. Besides, we identified eight nonhuman primate *POTE* sequences by cytogenetic assays that are not annotated in the corresponding reference genomes. Regions harboring gene families and SDs are prone to be misassembled and need more concerted efforts to be accurately completed.

Currently, with the advent of new sequencing technologies allowing high-throughput data generation, genome assemblies change quickly, thus generating the need for constant updates of gene annotations. This is particularly relevant for genes mapping to SDs where misassemblies are common due to the high sequence similarity between paralogous sequences. The *POTE* gene family represents a clear example, which required additional care to resolve the organization and copy number in human and nonhuman primates. Further additional sequencing efforts may disclose other copies in nonhuman primates that are missing or are collapsed in the current reference genomes. Combining SMRT sequencing, molecular cytogenetics, and long-read sequence analyses, we were able to shed light on the complex evolution of the *POTE* gene family, putting forward a complex mechanism involving pericentromeric exchange, gene conversion, LINE retrotransposition, and chromosomal rearrangement.

## Figures and Tables

**Table 1:** Members of the human *POTE* (prostate, ovary, testis, and placenta expressed) gene family.

HGNC ID	Approved Symbol	Approved Name	Ensembl ID	Synonyms	Genomic Localization	Gene Size (bp)	Transcript Variants
33893	*POTEA*	*POTE* ankyrin domain family member A	ENSG00000188877	*POTE8*, *POTE-8*, *CT104.3*	8p11.1	71,036	2
33734	*POTEB*	*POTE* ankyrin domain family member B	ENSG00000233917	*POTE15*, *POTE-15*, *CT104.5*	15q11.2	31,375	4
48327	*POTEB2*	*POTE* ankyrin domain family member B2	ENSG00000230031		15q11.2	30,943	2
51240	*POTEB3*	*POTE* ankyrin domain family member B3	ENSG00000278522		15q11.2	32,209	3
33894	*POTEC*	*POTE* ankyrin domain family member C	ENSG00000183206	*POTE18*, *POTE-18*, *DKFZp686J0529*, *CT104.6*	18p11.21	36,247	4
23822	*POTED*	*POTE* ankyrin domain family member D	ENSG00000166351	*POTE*, *POTE-21*, *POTE21*, *CT104.1*	21q11.2	31,728	2
33895	*POTEE*	*POTE* ankyrin domain family member E	ENSG00000188219	*POTE2*, *POTE-2*, *A26C1*, *POTE2gamma*, *CT104.2*	2q21.1	47,212	5
33905	*POTEF*	*POTE* ankyrin domain family member F	ENSG00000196604	*POTEACTIN*, *POTE2alpha*	2q21.1	55,688	2
33896	*POTEG*	*POTE* ankyrin domain family member G	ENSG00000187537	*POTE14*, *POTE-14*, *POTE14alpha*, *CT104.4*	14q11.2	31,856	3
133	*POTEH*	*POTE* ankyrin domain family member H	ENSG00000198062	*POTE22*, *CT104.7*	22q11.1	31,606	3
37093	*POTEI*	*POTE* ankyrin domain family member I	ENSG00000196834	*POTE2beta*	2q21.1	50,253	3
37094	*POTEJ*	*POTE* ankyrin domain family member J	ENSG00000222038	*POTE2beta*	2q21.1	46,611	1
30182	*POTEKP*	*POTE* ankyrin domain family member K, pseudogene	ENSG00000204434	*FKSG30*, *POTE2delta*	2q21.1	36,396	NA
37096	*POTEM*	*POTE* ankyrin domain family member M	ENSG00000222036	*POTE14beta*, *P704P*, *ACT*	14q11.2	36,319	3

**Table 2:** Human *POTE* gene features and domains.

Ensembl Gene ID	Approved SYMBOL	Gene Exons #	LIR	β-Actin Retrogene	Cysteine Repeats #	Ankyrin Repeats #	Coiled Coils #
**Group1**							
ENSG00000188877	*POTEA*	14	half (low similarity)	no	1	7 *	4
**Group2**							
ENSG00000233917	*POTEB*	11	half	no	2	7 *	1
ENSG00000230031	*POTEB2*	11	half	no	2	7 *	1
ENSG00000278522	*POTEB3*	11	half	no	3	7 *	1
ENSG00000183206	*POTEC*	11	half	no	3	7 *	1
ENSG00000166351	*POTED*	11	half	no	3	7 *	2
**Group3**							
ENSG00000188219	*POTEE*	15	full	yes	3	7 *	1
ENSG00000196604	*POTEF*	17	full	yes	3	7 *	1
ENSG00000196834	*POTEI*	15	full	yes	3	7 *	1
ENSG00000222038	*POTEJ*	15	full	yes	2	7 *	1
ENSG00000204434	*POTEKP*	11	full	yes (out of the CDS)	NA	NA	NA
**Group4**							
ENSG00000187537	*POTEG*	12	full	yes (out of the CDS)	3	7 *	0
ENSG00000198062	*POTEH*	12	full	yes (out of the CDS)	3–4	7 *	0
ENSG00000222036	*POTEM*	12	full	yes (out of the CDS)	3	7 *	0

* The prediction score of the first ankyrin is lower than the cutoffs established by SMART. #, numbers of elements.

**Table 3:** Primate *POTE* genes and corresponding transcript features and domains.

Species/Assembly	Ensembl Gene ID, Approved Symbol	Retrieved as Ortholog of Human	Mapping	Gene Exons #	Ensemble Transcript IDs	LIR	β-Actin Retrogene Position (aa)	Cysteine Repeats #	Ankyrin Repeats #	Coiled Coils #
**Chimpanzee (Pan troglodytes)/Pan_tro 3.0/panTro5**	ENSPTRG00000022971, *POTEA*	*POTEA*	8p (HSA8p)	12	ENSPTRT00000042301.5	no ^1^	no	1	7	1
	ENSPTRG00000006815, *POTEB*	Group II genes	15q (HSA15q)	11	ENSPTRT00000042433.5	half	no	3	7	1
					ENSPTRT00000100986.1	no	no	3	7	0
	ENSPTRG00000022897	*POTEE/F*	2Bp (HSA2q)	16	ENSPTRT00000037475.5	full	708-1078	3	7	1
					ENSPTRT00000050738.5	no	no	3	7	0
	ENSPTRG00000024151	*POTEI/POTEJ*	2Bp (HSA2q)	13	ENSPTRT00000092585.1	no	no	3	7	0
					ENSPTRT00000089094.1	no	no	3	7	0
					ENSPTRT00000078184.1	no	no	3	7	0
	ENSPTRG00000050347	Group IV genes	14q (HSA14q)	8	ENSPTRT00000092958.1	full ^1^	NA	3	0	0
**Gorilla (Gorilla gorilla gorilla)/gorGor4.1/gorGor4**	ENSGGOG00000028525	*POTEA*	8q (HSA8p)	11	ENSGGOT00000024046.2	no	NA	1	7	1
	ENSGGOG00000015216	Group II genes	15 (HSA15q)	7	ENSGGOT00000015273.3	half ^1^	NA	1	7	0
	ENSGGOG00000007645	Group III genes	2Bq (HSA2q)	9	ENSGGOT00000029469.2	full ^1^	yes ^1^	1	5	0
**Orangutan (Pongo abelii)/WUGSC 2.0.2/ponAbe2**	ENSPPYG00000018567, *POTEA*	*POTEA*	8 (HSA8p)	14	ENSPPYT00000021656.1	half	no	1	7	2
	ENSPPYG00000019944	Group II genes	Un (HSA15q)	5	ENSPPYT00000023266.1	NA	no	3	5	0
					ENSPPYT00000023267.1	NA	no	2	5	0
	ENSPPYG00000005548	Group III genes	14 (HSA14q)	11	ENSPPYT00000006570.1	full ^1^	yes ^1^	0	5	0
	ENSPPYG00000012746	Group IV genes	2B (HSA2q)	11	ENSPPYT00000014802.1	full	yes ^1^	3	6	1
**Macaque (Macaca mulatta)/BCM Mmul_8.0.1/rheMac8**	ENSMMUG00000031128	*POTEA* ^2^	10q (HSA20)	7	ENSMMUT00000044360.3	no	NA	3	7	0
	ENSMMUG00000001813	Group III-IV genes	10q (HSA20)	6	ENSMMUT00000047624.3	NA	NA	2	5	0
	ENSMMUG00000041125	Group III-IV genes	12 (HSA2q)	6	ENSMMUT00000044359.3	no	NA	1	6	0
**Marmoset (Callithrix jacchus)/ASM275486v1**	ENSCJAG00000033335	Group II-III-IV genes	Contig NTIC01007779.1	7	ENSCJAT00000063391.3	NA	NA	1	7	0
					ENSCJAT00000098614.1	NA	NA	1	6	0
	CH259-195P19_g1 ^3^	NA	16p (HSA8p)	9 ^4^	NA	half	no	1	5	2
	CH259-195P19_g2 ^3^	NA	16p (HSA8p)	4 ^4^	NA	NA	NA	1	4	0

NA = not assessed; #, numbers of elements; ^1^ the primate gene does not contain this region, but the flanking region in the reference genome does, and it is embedded in the human *POTE* RefSeq; ^2^ this gene is not listed as an ortholog of human *POTEA*, but shares the same Ensembl gene tree (ENSGT00940000164614); ^3^ these genes have been retrieved through a high-density filters screening of a marmoset BAC library; ^4^ as predicted by Augustus.

**Table 4:** FISH results obtained from hybridizing human fosmid clones containing *POTE* sequences (ABC12-49207800H13, ABC12-49059300N17, ABC12-46762300H6, ABC8-41159000G3) from human, chimpanzee, gorilla, orangutan, and macaque metaphases. Classical karyotype nomenclature is used for PTR, GGO, and PPY; MMU nomenclature according to Rogers et al. [42].

	HSA2p	HSA2q	HSA8	HSA10	HSA13	HSA14	HSA15	HSA18	HSA20	HSA21	HSA22
**Human**		yes	yes ^1^			yes ^2^	yes ^2^	yes ^2^		yes ^2^	yes ^2^
**Chimpanzee**		yes (PTR13)	yes (PTR7) ^1^			yes (PTR15) ^2^	yes (PTR16) ^2^	yes (PTR17) ^2^		yes (PTR22) ^2^	
**Gorilla**		yes (GGO11) ^2^	yes (GGO7) ^1^		yes (GGO14) ^3^	yes (GGO18) ^2^	yes (GGO15) ^2^	yes (GGO16) ^2^			
**Orangutan**	yes (PPY12) ^2^	yes (PPY11) ^2^	yes (PPY6) ^1^			yes (PPY15) ^2^	yes (PPY16) ^2^	yes (PPY17) ^2^		yes (PPY 22) ^2^	
**Macaque**		yes (MMU12) ^2^		yes (MMU9) ^2^					yes (MMU10) ^2^		

^1^ only with probe ABC12-49207800H13, ^2^ with all probes except ABC12-49207800H13, ^3^ only with probe ABC12-49059300N17.

## Supplementary Materials

The following are available online at https://www.mdpi.com/2073-4425/11/2/213/s1, Figure S1: Domain structure of the canonical (underlined) and alternative transcripts of human *POTE* genes; Figure S2: Domain structure of all annotated and predicted transcripts of the studied primate *POTE* genes; Figure S3: Gene prediction by Augustus; Figure S4: Phylogenetic analysis of group III intragenic duplication; Figure S5: LINE contents in primates; Figure S6: Evolutionary analyses of human-specific *POTE* gene duplications; Table S1: Human *POTE* genes, canonical, and alternative transcript features and domains, with positions; Table S2: Log2fold change values in cancer vs. normal tissues; Table S3: Primate *POTE* genes and corresponding transcript features and domains, with positions; Table S4: *Callithrix jacchus* BAC clones positive with respect to the BAC library screening and PCR with primer pairs for STS1 and STS2; Table S5: *POTE* genes predicted by Augustus in the PacBio-sequenced marmoset BAC clones.

## Data Access

Assembled sequencing data from PacBio SMRTbell sequencing experiments from this study have been submitted to the BankIt with the IDs MN822058, MN822059, MN822060, and MN822061.

## References

[1] Alkan C., Kidd J.M., Marques-Bonet T., Aksay G., Antonacci F., Hormozdiari F., Kitzman J.O., Baker C., Malig M., Mutlu O. (2009). Personalized copy number and segmental duplication maps using next-generation sequencing. Nat. Genet..

[2] Bailey J.A., Yavor A.M., Massa H.F., Trask B.J., Eichler E.E. (2001). Segmental duplications: Organization and impact within the current human genome project assembly. Genome Res..

[3] Iafrate A.J., Feuk L., Rivera M.N., Listewnik M.L., Donahoe P.K., Qi Y., Scherer S.W., Lee C. (2004). Detection of large-scale variation in the human genome. Nat. Genet..

[4] Itsara A., Cooper G.M., Baker C., Girirajan S., Li J., Absher D., Krauss R.M., Myers R.M., Ridker P.M., Chasman D.I. (2009). Population analysis of large copy number variants and hotspots of human genetic disease. Am. J. Hum. Genet..

[5] Catacchio C.R., Maggiolini F.A.M., D’Addabbo P., Bitonto M., Capozzi O., Lepore Signorile M., Miroballo M., Archidiacono N., Eichler E.E., Ventura M. (2018). Inversion variants in human and primate genomes. Genome Res..

[6] Maggiolini F.A.M., Cantsilieris S., D’Addabbo P., Manganelli M., Coe B.P., Dumont B.L., Sanders A.D., Pang A.W.C., Vollger M.R., Palumbo O. (2019). Genomic inversions and GOLGA core duplicons underlie disease instability at the 15q25 locus. PLoS Genet..

[7] Dennis M.Y., Eichler E.E. (2016). Human adaptation and evolution by segmental duplication. Curr. Opin. Genet. Dev..

[8] Dennis M.Y., Nuttle X., Sudmant P.H., Antonacci F., Graves T.A., Nefedov M., Rosenfeld J.A., Sajjadian S., Malig M., Kotkiewicz H. (2012). Evolution of human-specific neural *SRGAP2* genes by incomplete segmental duplication. Cell.

[9] Giannuzzi G., Siswara P., Malig M., Marques-Bonet T., Mullikin J.C., Ventura M., Eichler E.E., Program N.C.S. (2013). Evolutionary dynamism of the primate *LRRC37* gene family. Genome Res..

[10] Suzuki I.K., Gacquer D., Van Heurck R., Kumar D., Wojno M., Bilheu A., Herpoel A., Lambert N., Cheron J., Polleux F. (2018). Human-specific *NOTCH2NL* genes expand cortical neurogenesis through delta/notch regulation. Cell.

[11] Fiddes I.T., Pollen A.A., Davis J.M., Sikela J.M. (2019). Paired involvement of human-specific Olduvai domains and *NOTCH2NL* genes in human brain evolution. Hum. Genet..

[12] Simpson A.J., Caballero O.L., Jungbluth A., Chen Y.T., Old L.J. (2005). Cancer/testis antigens, gametogenesis and cancer. Nat. Rev. Cancer.

[13] Coulie P.G., Van den Eynde B.J., van der Bruggen P., Boon T. (2014). Tumour antigens recognized by T lymphocytes: At the core of cancer immunotherapy. Nat. Rev. Cancer.

[14] Sharma A., Albahrani M., Zhang W., Kufel C.N., James S.R., Odunsi K., Klinkebiel D., Karpf A.R. (2019). Epigenetic activation of *POTE* genes in ovarian cancer. Epigenetics.

[15] Bera T.K., Zimonjic D.B., Popescu N.C., Sathyanarayana B.K., Kumar V., Lee B., Pastan I. (2002). *POTE*, a highly homologous gene family located on numerous chromosomes and expressed in prostate, ovary, testis, placenta, and prostate cancer. Proc. Natl. Acad. Sci. USA.

[16] Barger C.J., Zhang W., Sharma A., Chee L., James S.R., Kufel C.N., Miller A., Meza J., Drapkin R., Odunsi K. (2018). Expression of the *POTE* gene family in human ovarian cancer. Sci. Rep..

[17] Bera T.K., Saint Fleur A., Lee Y., Kydd A., Hahn Y., Popescu N.C., Zimonjic D.B., Lee B., Pastan I. (2006). POTE paralogs are induced and differentially expressed in many cancers. Cancer Res..

[18] Redfield S.M., Mao J., Zhu H., He Z., Zhang X., Bigler S.A., Zhou X. (2013). The C-terminal common to group 3 POTES (CtG3P): A newly discovered nucleolar marker associated with malignant progression and metastasis. Am. J. Cancer Res..

[19] Bera T.K., Saint Fleur A., Ha D., Yamada M., Lee Y., Lee B., Hahn Y., Kaufman D.S., Pera M., Pastan I. (2008). Selective POTE paralogs on chromosome 2 are expressed in human embryonic stem cells. Stem Cells Dev..

[20] Hahn Y., Bera T.K., Pastan I.H., Lee B. (2006). Duplication and extensive remodeling shaped *POTE* family genes encoding proteins containing ankyrin repeat and coiled coil domains. Gene.

[21] Giannuzzi G., Pazienza M., Huddleston J., Antonacci F., Malig M., Vives L., Eichler E.E., Ventura M. (2013). Hominoid fission of chromosome 14/15 and the role of segmental duplications. Genome Res..

[22] Lee Y., Ise T., Ha D., Saint Fleur A., Hahn Y., Liu X.F., Nagata S., Lee B., Bera T.K., Pastan I. (2006). Evolution and expression of chimeric *POTE*-actin genes in the human genome. Proc. Natl. Acad. Sci. USA.

[23] Bera T.K., Huynh N., Maeda H., Sathyanarayana B.K., Lee B., Pastan I. (2004). Five POTE paralogs and their splice variants are expressed in human prostate and encode proteins of different lengths. Gene.

[24] Wang Y., Leung F.C. (2009). Discovery of a long inverted repeat in human *POTE* genes. Genomics.

[25] Kent W.J. (2002). BLAT—The BLAST-like alignment tool. Genome Res..

[26] Lichter P., Jauch A., Cremer T., Ward D.C. (1990). Detection of Down syndrome by in situ hybridization with chromosome 21 specific DNA probes. Prog. Clin. Biol. Res..

[27] Vollger M.R., Logsdon G.A., Audano P.A., Sulovari A., Porubsky D., Peluso P., Wenger A.M., Concepcion G.T., Kronenberg Z.N., Munson K.M. (2019). Improved assembly and variant detection of a haploid human genome using single-molecule, high-fidelity long reads. Ann. Hum. Genet..

[28] Koren S., Walenz B.P., Berlin K., Miller J.R., Bergman N.H., Phillippy A.M. (2017). Canu: Scalable and accurate long-read assembly via adaptive. Genome Res..

[29] Tamura K., Battistuzzi F.U., Billing-Ross P., Murillo O., Filipski A., Kumar S. (2012). Estimating divergence times in large molecular phylogenies. Proc. Natl. Acad. Sci. USA.

[30] Holmquist R., Cantor C., Jukes T. (1972). Improved procedures for comparing homologous sequences in molecules of proteins and nucleic acids. J. Mol. Biol..

[31] Kumar S., Stecher G., Tamura K. (2016). MEGA7: Molecular evolutionary genetics analysis version 7.0 for bigger datasets. Mol. Biol. Evol..

[32] Bray N.L., Pimentel H., Melsted P., Pachter L. (2016). Near-optimal probabilistic RNA-seq quantification. Nat. Biotechnol..

[33] Pimentel H., Bray N.L., Puente S., Melsted P., Pachter L. (2017). Differential analysis of RNA-seq incorporating quantification uncertainty. Nat. Methods.

[34] Larkin M.A., Blackshields G., Brown N.P., Chenna R., McGettigan P.A., McWilliam H., Valentin F., Wallace I.M., Wilm A., Lopez R. (2007). Clustal W and Clustal X version 2.0. Bioinformatics.

[35] Yates B., Braschi B., Gray K.A., Seal R.L., Tweedie S., Bruford E.A. (2017). Genenames.org: The HGNC and VGNC resources in 2017. Nucleic Acids Res..

[36] Zerbino D.R., Achuthan P., Akanni W., Amode M.R., Barrell D., Bhai J., Billis K., Cummins C., Gall A., Girón C.G. (2018). Ensembl 2018. Nucleic Acids Res..

[37] Schultz J., Copley R.R., Doerks T., Ponting C.P., Bork P. (2000). SMART: A web-based tool for the study of genetically mobile domains. Nucleic Acids Res..

[38] Lupas A., Van Dyke M., Stock J. (1991). Predicting coiled coils from protein sequences. Science.

[39] Huddleston J., Chaisson M.J.P., Steinberg K.M., Warren W., Hoekzema K., Gordon D., Graves-Lindsay T.A., Munson K.M., Kronenberg Z.N., Vives L. (2017). Discovery and genotyping of structural variation from long-read haploid genome sequence data. Genome Res..

[40] Stanke M., Morgenstern B. (2005). AUGUSTUS: A web server for gene prediction in eukaryotes that allows user-defined constraints. Nucleic Acids Res..

[41] Khan H., Smit A., Boissinot S. (2006). Molecular evolution and tempo of amplification of human LINE-1 retrotransposons since the origin of primates. Genome Res..

[42] Rogers J., Garcia R., Shelledy W., Kaplan J., Arya A., Johnson Z., Bergstrom M., Novakowski L., Nair P., Vinson A. (2006). An initial genetic linkage map of the rhesus macaque (Macaca mulatta) genome using human microsatellite loci. Genomics.

[43] Marques-Bonet T., Kidd J.M., Ventura M., Graves T.A., Cheng Z., Hillier L.W., Jiang Z., Baker C., Malfavon-Borja R., Fulton L.A. (2009). A burst of segmental duplications in the genome of the African great ape ancestor. Nature.

[44] Consortium A., Marmoset G.S. (2014). The common marmoset genome provides insight into primate biology and evolution. Nat. Genet..

[45] Bailey J.A., Church D.M., Ventura M., Rocchi M., Eichler E.E. (2004). Analysis of segmental duplications and genome assembly in the mouse. Genome Res..

[46] Liu X.F., Bera T.K., Liu L.J., Pastan I. (2009). A primate-specific POTE-actin fusion protein plays a role in apoptosis. Apoptosis.

[47] Ventura M., Antonacci F., Cardone M.F., Stanyon R., D’Addabbo P., Cellamare A., Sprague L.J., Eichler E.E., Archidiacono N., Rocchi M. (2007). Evolutionary formation of new centromeres in macaque. Science.

[48] Yunis J.J., Prakash O. (1982). The origin of man: A chromosomal pictorial legacy. Science.

[49] Wienberg J., Jauch A., Lüdecke H.J., Senger G., Horsthemke B., Claussen U., Cremer T., Arnold N., Lengauer C. (1994). The origin of human chromosome 2 analyzed by comparative chromosome mapping with a DNA microlibrary. Chromosome Res..

[50] Archidiacono N., Antonacci R., Marzella R., Finelli P., Lonoce A., Rocchi M. (1995). Comparative mapping of human alphoid sequences in great apes using fluorescence in situ hybridization. Genomics.

[51] Alexandrov I.A., Mitkevich S.P., Yurov Y.B. (1988). The phylogeny of human chromosome specific alpha satellites. Chromosoma.

[52] Catacchio C.R., Ragone R., Chiatante G., Ventura M. (2015). Organization and evolution of Gorilla centromeric DNA from old strategies to new approaches. Sci. Rep..

[53] Ventura M., Mudge J.M., Palumbo V., Burn S., Blennow E., Pierluigi M., Giorda R., Zuffardi O., Archidiacono N., Jackson M.S. (2003). Neocentromeres in 15q24-26 map to duplicons which flanked an ancestral centromere in 15q25. Genome Res..

[54] Eichler E.E., Lu F., Shen Y., Antonacci R., Jurecic V., Doggett N.A., Moyzis R.K., Baldini A., Gibbs R.A., Nelson D.L. (1996). Duplication of a gene-rich cluster between 16p11.1 and Xq28: A novel pericentromeric-directed mechanism for paralogous genome evolution. Hum. Mol. Genet..

[55] Vandepoele K., Van Roy N., Staes K., Speleman F., van Roy F. (2005). A novel gene family *NBPF*: Intricate structure generated by gene duplications during primate evolution. Mol. Biol. Evol..

[56] Johnson M.E., Viggiano L., Bailey J.A., Abdul-Rauf M., Goodwin G., Rocchi M., Eichler E.E. (2001). Positive selection of a gene family during the emergence of humans and African apes. Nature.

[57] Eichler E.E., Budarf M.L., Rocchi M., Deaven L.L., Doggett N.A., Baldini A., Nelson D.L., Mohrenweiser H.W. (1997). Interchromosomal duplications of the adrenoleukodystrophy locus: A phenomenon of pericentromeric plasticity. Hum. Mol. Genet..

[58] Kumar S., Stecher G., Suleski M., Hedges S.B. (2017). TimeTree: A resource for timelines, timetrees, and divergence times. Mol. Biol. Evol..

[59] Nei M.s, Rooney A.P. (2005). Concerted and birth-and-death evolution of multigene families. Annu. Rev. Genet..

